# Proliferative diabetic retinopathy transcriptomes reveal angiogenesis, anti-angiogenic therapy escape mechanisms, fibrosis and lymphatic involvement

**DOI:** 10.1038/s41598-021-97970-5

**Published:** 2021-09-22

**Authors:** Ani Korhonen, Erika Gucciardo, Kaisa Lehti, Sirpa Loukovaara

**Affiliations:** 1 Individualized Drug Therapy Research Program, Faculty of Medicine, University of Helsinki, Helsinki, Finland; 2 Department of Microbiology, Tumor, and Cell Biology (MTC), Karolinska Institutet, Stockholm, Sweden; 3 Department of Biomedical Laboratory Science, Norwegian University of Science and Technology, Trondheim, Norway; 4 Unit of Vitreoretinal Surgery, Ophthalmology, University of Helsinki and Helsinki University Hospital, Helsinki, Finland

**Keywords:** Retinal diseases, RNA sequencing, Cellular signalling networks, Angiogenesis, Transcriptomics

## Abstract

Proliferative diabetic retinopathy (PDR) is a sight-threatening diabetic complication in urgent need of new therapies. In this study we identify potential molecular mechanisms and target candidates in the pathogenesis of PDR fibrovascular tissue formation. We performed mRNA sequencing of RNA isolated from eleven excised fibrovascular membranes of type 1 diabetic PDR patients and two non-diabetic patients with rhegmatogenous retinal detachment with proliferative vitreoretinopathy. We determined differentially expressed genes between these groups and performed pathway and gene ontology term enrichment analyses to identify potential underlying mechanisms, pathways, and regulators. Multiple pro-angiogenic processes, including VEGFA-dependent and -independent pathways, as well as processes related to lymphatic development, epithelial to mesenchymal transition (EMT), wound healing, inflammation, fibrosis, and extracellular matrix (ECM) composition, were overrepresented in PDR. Overrepresentation of different angiogenic processes may help to explain the transient nature of the benefits that many patients receive from current intravitreal anti-angiogenic therapies, highlighting the importance of combinatorial treatments. Enrichment of genes and pathways related to lymphatic development indicates that targeting lymphatic involvement in PDR progression could have therapeutic relevance. Together with overrepresentation of EMT and fibrosis as well as differential ECM composition, these findings demonstrate the complexity of PDR fibrovascular tissue formation and provide avenues for the development of novel treatments.

## Introduction

Diabetic retinopathy (DR) is a sight-threatening microvascular complication of diabetes, developing in nearly all patients with type 1 diabetes (T1D), and in majority of patients with T2D^1^. Proliferative DR (PDR) is the end-stage disease, with hypoxia-induced pathological neovascularization at the vitreoretinal interface, often accompanied by vitreous haemorrhage (VH), tractional retinal detachment (TRD) and diabetic macular oedema (DME). PDR progression involves a complex interplay of biochemical, immunological and inflammatory factors, but the exact mechanisms remain unclear^2^.

Panretinal photocoagulation (PRP) and intravitreal anti-VEGFA injections are used to treat DR complications, but often to poor or transient effects. Vitrectomy is performed in cases of prolonged VH and/or TRD, and of combined tractional and rhegmatogenous retinal detachment. Microarray analysis has previously revealed gene expression differences in PDR tissues compared to normal human retina^3^, but global RNA expression of PDR tissues by sequencing has not been investigated.

Rhegmatogenous retinal detachment (RRD), detachment of the neurosensory retina from the retinal pigment epithelium (RPE), occurs when fluid enters and accumulates in the subretinal space after e.g. retinal tears upon trauma. 5–11% of RRD cases develop proliferative vitreoretinopathy (PVR)^4^, abnormal wound healing where proliferative and contractile membranes form on the retina upon ischemia, cell death and blood-retinal barrier breakdown due to RRD. RPE cells and glial cells are major contributors to these membranes, but exact pathological mechanisms remain unclear^5^. RPE cells undergo epithelial-to-mesenchymal transition (EMT), though other cell types like hyalocytes and glial cells also take part in extracellular matrix (ECM) deposition and fibrosis^6^.

We here performed bulk RNA sequencing to determine gene expression patterns in PDR fibrovascular tissues and avascular fibrous RRD-PVR tissues in order to better understand the mechanisms of these sight-threatening diseases and identify potential therapeutic targets.

## Results

### Differential gene expression between proliferative diabetic retinopathy and rhegmatogenous retinal detachment with proliferative vitreoretinopathy

**Figure 1:**
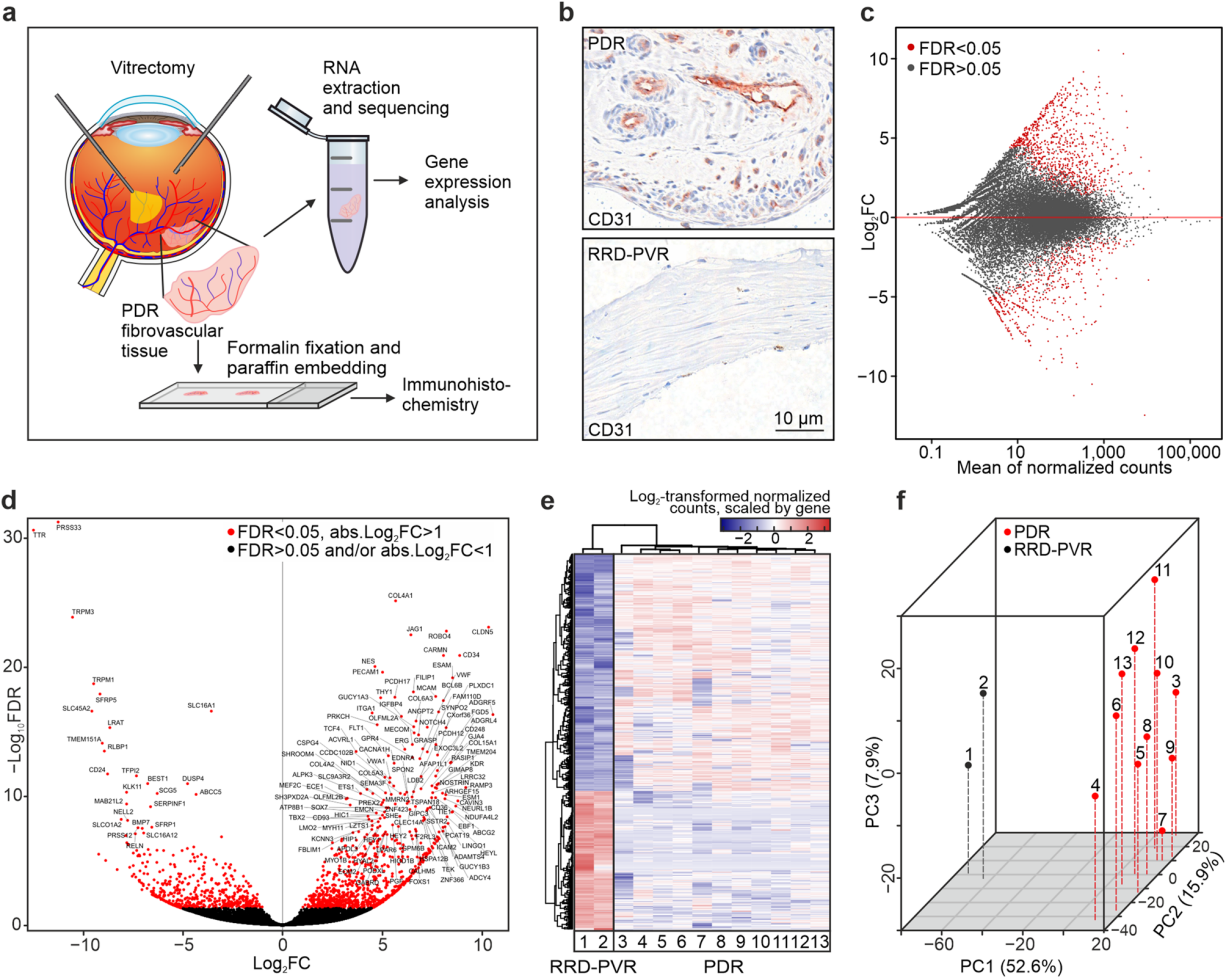
Overview of RNA-sequencing. (**a**) Schematic workflow: the PDR and RRD-PVR tissues were removed during vitrectomy. Excised tissues were either placed in RNA-later, processed for sequencing and sequenced; or formalin fixed, paraffin embedded (FFPE) and cut into sections for immunostaining. From sequencing data, differentially expressed genes (DEGs) were identified, and enrichment analysis was performed to identify overrepresented pathways, molecular functions, biological processes, and cellular components. (**b**) Representative light micrographs of immunohistochemistry for CD31 visualizes endothelial vascular structures in PDR and absence of vasculature in RRD-PVR tissue. Scale bar, 10 µm. (**c**) MA-plot of log_2_-fold change and mean normalized count for all genes. Each gene is represented by a grey dot. Differentially expressed genes (DEGs) are indicated in red. (**d**) Volcano plot of gene expression. (**e**) Heatmap representing color-coded expression levels of differentially expressed genes. Hierarchical clustering analysis performed using log-transformed normalized read counts of DEGs (FDR-corrected *p* value < 0.05, absolute log_2_fold change > 1) between RRD-PVR patients (n = 2) and PDR patients (n = 11), showing interpatient variability. (**f**) 3D principal component analysis (PCA) plot with PDR (red) and RRD-PVR (black) samples plotted in three dimensions using their projection onto the first three principal components (PC1, PC2, PC3). Each dot represents a patient. Principal component analysis was performed using the 500 most variable genes. PC1, PC2 and PC3 explained 52.6%, 15.9% and 7.9% of the variation, respectively. The PDR samples clearly segregated from the RRD-PVR group along PC1 and showed more variability on PC2 and PC3.

To analyse gene expression in PDR, we sequenced mRNA isolated from fibrovascular membranes of PDR patients (n = 11) and mRNA from avascular fibrotic tissues of non-diabetic RRD-PVR patients (n = 2) and compared their transcriptomes (Fig. 1A). The PDR tissues present neovascularization, whereas the RRD-PVR tissues show fibrosis without vasculature (Fig. 1B). See Supplementary Table S1 for summarized and Supplementary Table S2 for individual patients’ systemic and ocular characteristics.

Out of 26,621 genes with non-zero total read count, 1447 were significantly differentially expressed genes (DEGs, FDR < 0.05 and absolute log_2_-fold change > 1). Of these, 910 were upregulated and 537 downregulated in the PDR cohort (Fig. 1C,D, Supplementary Table S3). Hierarchical clustering based on the DEGs resulted in segregation of PDR from RRD-PVR patients and revealed interpatient variation (Fig. 1E).

When projecting the PDR and RRD-PVR patients on a two-dimensional space after principal component (PC) analysis, the PDR patients segregated from the RRD-PVR patients along PC1 which explained 52.6% of the variation (Fig. 1F). Gene ontology (GO) enrichment analysis (GOEA) of the top 50 PC1 genes revealed vasculature-related biological processes (Supplementary Table S4), indicating that presence/absence of vasculature distinguished the patients, as neovascularization occurs in PDR and not in RRD-PVR. PC2 and PC3 explained 15.9% and 7.9% of the variation, respectively, and the patient groups did not segregate along these PCs (Fig. 1F, Supplementary Table S4).

### Angiogenesis, inflammation, and fibrosis are overrepresented in proliferative diabetic retinopathy

**Figure 2:**
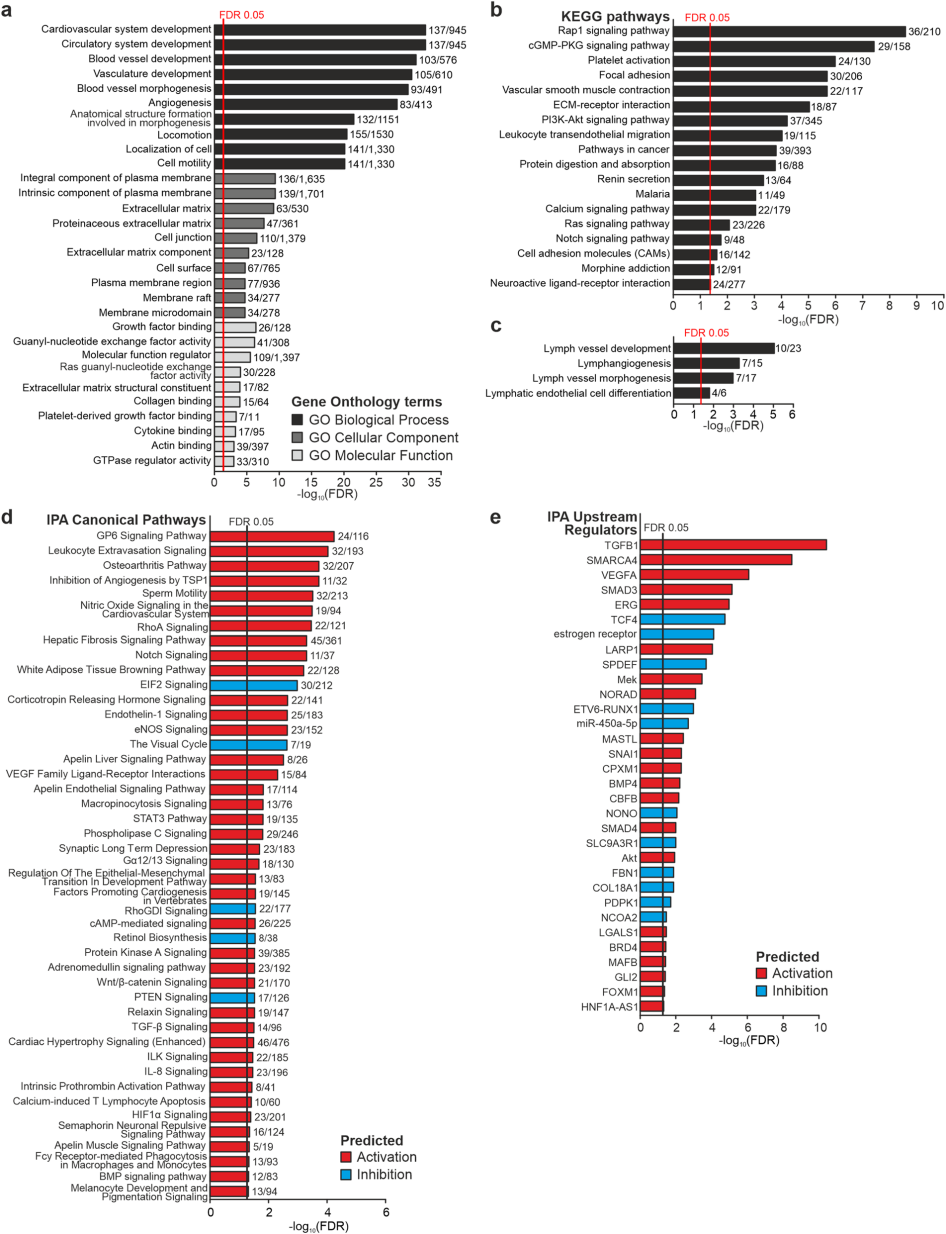
Gene ontology (GO)-term, pathway enrichment as well as IPA analyses reveal involvement of angiogenesis, inflammation, and fibrosis in proliferative diabetic retinopathy. (**a**) Bar graph showing top 10 most significant Gene Ontology (GO) biological processes, cellular components and molecular functions significantly enriched based on DEGs upregulated in PDR (see Supplementary Table S5 for full list of enriched GO-terms). Numbers beside bars indicate the number of significant genes in the GO-term versus the total number of genes in the GO-term. (**b**) Bar graph showing Kyoto Encyclopaedia of Genes and Genomes (KEGG) pathways overrepresented based on DEGs upregulated in PDR (see Supplementary Table S5D for full list of enriched KEGG pathways). Numbers beside bars indicate the number of significant genes in the pathway versus the total number of genes in the pathway. (**c**) Bar graph showing GO-terms related to lymphatics, significantly enriched based on DEGs upregulated in PDR. Numbers beside bars indicate the number of significant genes in the GO-term versus the total number of genes in the GO-term. (**d**) Bar graph showing significantly overrepresented Ingenuity Pathway Analysis (IPA) canonical pathways predicted to be activated (red) or inhibited (azure), FDR < 0.05. Numbers beside bars indicate number of molecules meeting the cut-off/molecules in the pathway versus the total number of molecules in the pathway. (**e**) Bar graph showing significantly overrepresented IPA Upstream regulators predicted to be activated (red) or inhibited (azure), FDR < 0.05.

The relative RNA expression between PDR and RRD-PVR allows investigation into the commonalities between these diseases, including fibrosis and inflammation, as well as into the PDR-specific processes. Based on the DEGs upregulated in PDR, 701 biological processes (BPs), 53 cellular components (CCs) and 37 molecular functions (MFs) were significant in GOEA (Fig. 2A, Supplementary Table S5A-C), as were 18 KEGG pathways (Fig. 2B, Supplementary Table S5D). The top ten BPs were related to angiogenesis, vasculature development and cell motility (Fig. 2A). Notably, *Lymph vessel development, Lymphangiogenesis, Lymph vessel morphogenesis* and *Lymphatic endothelial cell differentiation* were also significant (Fig. 2C, Supplementary Table S5A). Enriched CCs were related to ECM, plasma membrane, stress fibres, cell junctions and cell leading edge. Enriched MFs included growth factor, actin, collagen, and cytokine binding (Fig. 2A, Supplementary Table S5B-C). KEGG pathways related to ECM and cytoskeleton such as *Rap1 signalling* and *Focal adhesion*, as well as pathways *Platelet activation, Leukocyte transendothelial migration* and *Vascular smooth muscle contraction* were also significant (Fig. 2C, Supplementary Table S5D).

Ingenuity Pathway Analysis (IPA) of all DEGs identified 40 significantly activated IPA canonical pathways (Fig. 2D, Supplementary Table S6A). These included *VEGF Family Ligand-Receptor Interactions* and *Nitric Oxide Signalling in the Cardiovascular System*, pathways involved in inflammation and wound healing, such as *Leukocyte Extravasation Signalling*, *IL-8 Signalling* and *STAT3 Pathway*, and EMT-related pathways such as *Regulation of the EMT in Development Pathway*. In addition to pro-angiogenic pathways, *Inhibition of Angiogenesis by Thrombospondin 1 (TSP1)* was predicted to be activated (Supplementary Fig. S1A). Pathways predicted to be inhibited included *EIF2 Signalling and the Visual Cycle* (Supplementary Fig. S1B, Supplementary Table S6A). Other significantly altered pathways were related to fibrosis, such as *Hepatic Fibrosis/Hepatic Stellate Cell Activation,* and to inflammation, such as *Granulocyte Adhesion and Diapedesis* and *Agranulocyte Adhesion and Diapedesis* (Supplementary Fig. S1C).

To investigate potential factors regulating the observed gene expression changes, we performed IPA upstream regulator analysis (URA) and identified 21 activated upstream regulators (URs) (Z-score > 2; Fig. 2E, Supplementary Table S6B). The top activated URs were transforming growth factor-β1 (TGF-β1), transcription regulator SWI/SNF related, matrix associated, actin dependent regulator of chromatin, subfamily a, member 4 (SMARCA4), vascular endothelial growth factor-A (VEGFA), the TGF-β and activin downstream transcription regulator SMAD3, and regulator of endothelial cell (EC) function, erythroblast transformation-specific (ETS) -related gene (ERG). Eleven URs were predicted to be inhibited, including TCF4, estrogen receptor and SPDEF (Z-score < -2; Fig. 2E, Supplementary Table S6B). Altogether, these findings implicate vascularization, fibrosis, and inflammation in PDR pathophysiology.

### Angiogenesis through VEGFA-ligand-dependent mechanisms is involved in proliferative diabetic retinopathy

**Figure 3:**
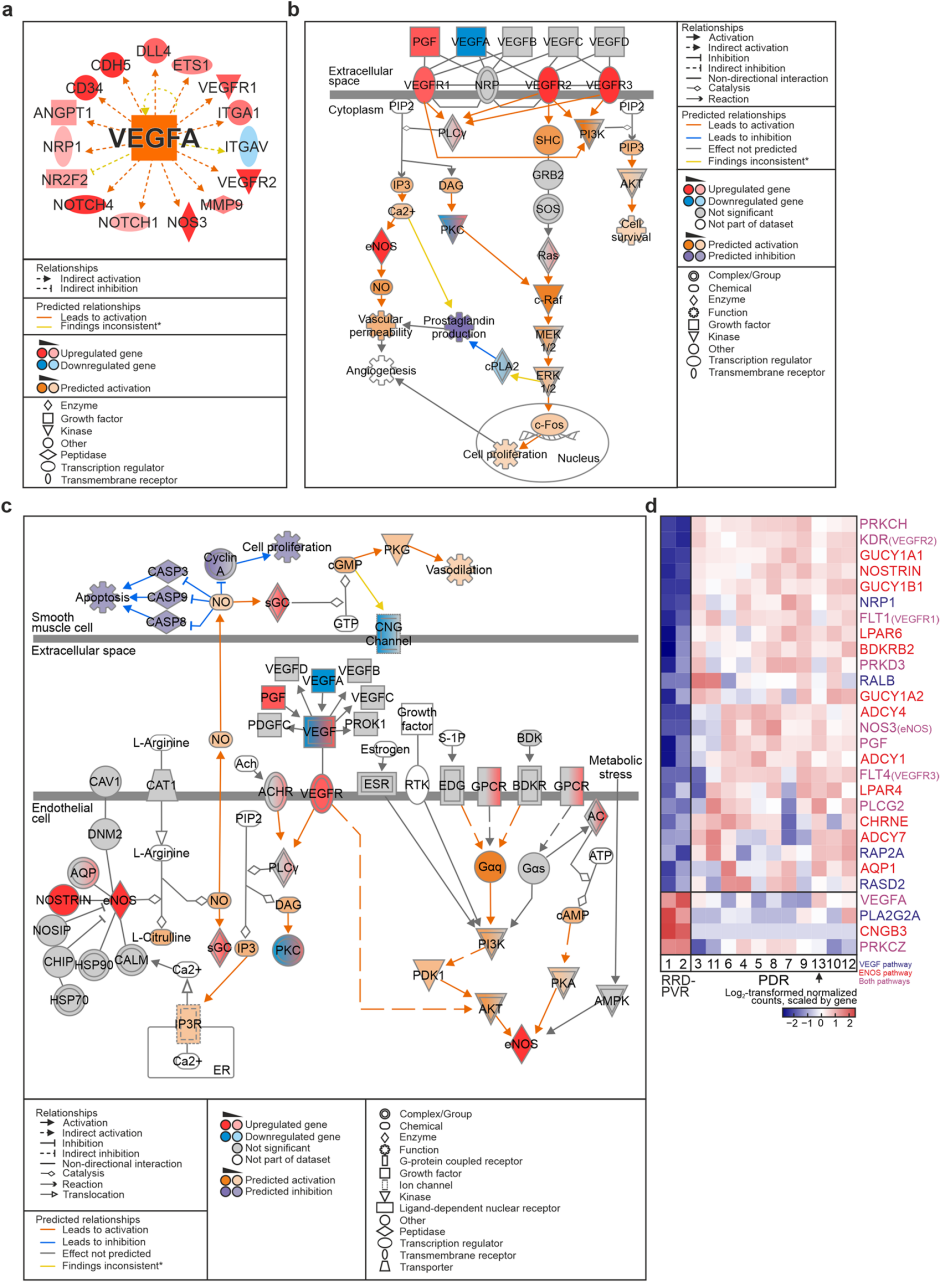
VEGFA-ligand-dependent mechanisms are involved in proliferative diabetic retinopathy. (**a**) Network of DEGs affected by VEGFA (Ingenuity Pathway Analysis (IPA)), based on which VEGFA was predicted as an activated upstream regulator. (**b**) IPA canonical pathway *VEGF family ligand receptor interactions.* (**c**) IPA canonical pathway *Enos signalling*. (**d**) Heatmap of log_2_-transformed normalized read counts of DEGs in *VEGF family ligand receptor interactions* and *Enos signalling* IPA canonical pathways. Genes in *VEGF family ligand receptor interactions*, *Enos signalling* or both pathways are indicated in blue, red, and purple, respectively. Arrow indicates the PDR patient who had received no preoperative retinal laser photocoagulation (Patient 13). (*) Yellow arrows in (**b**) and (**d**) indicate that findings are inconsistent with the state of downstream molecule (IPA). The network and pathways in (**a**), (**b**) and (**c**) were generated through the use of IPA (QIAGEN Inc., https://www.qiagenbioinformatics.com/products/ingenuity-pathway-analysis)^45^.

To understand the potential involvement of different pro-angiogenic mechanisms in PDR, we examined the VEGFA-dependent pathways and interactions identified by IPA. VEGFA, main proangiogenic factor involved in PDR, was predicted an activated UR (Fig. 3A). *VEGF Family Ligand-Receptor Interactions* (Fig. 3B) and closely related *Endothelial nitric oxide synthase (eNOS, NOS3) signalling* pathways were also predicted to be activated (Fig. 3B-C). Fifteen of 84 genes included in *VEGF Family Ligand-Receptor Interactions*, and 23 of 152 genes in *eNOS signalling* were DEGs (Fig. 3D). VEGFA activates eNOS via Akt-pathway, and eNOS regulates EC function and blood vessel maturation in retinal angiogenesis^7^. Consistently, Akt was predicted an activated UR (Fig. 2E). VEGFA receptors VEGFR2 (KDR) and VEGFR1 (FLT1) were upregulated, as was co-receptor NRP1. Although found at higher levels in RRD-PVR than PDR, VEGFA was expressed across all PDR tissues (Fig. 3D). Moreover, within the PDR group, VEGFA-expression was highest in the patient who had received no preoperative retinal laser photocoagulation (Fig. 3D). Altogether, these results are consistent with involvement of VEGFA-ligand-dependent mechanisms in PDR.

### VEGFA-ligand-independent mechanisms may contribute to angiogenesis in PDR

**Figure 4:**
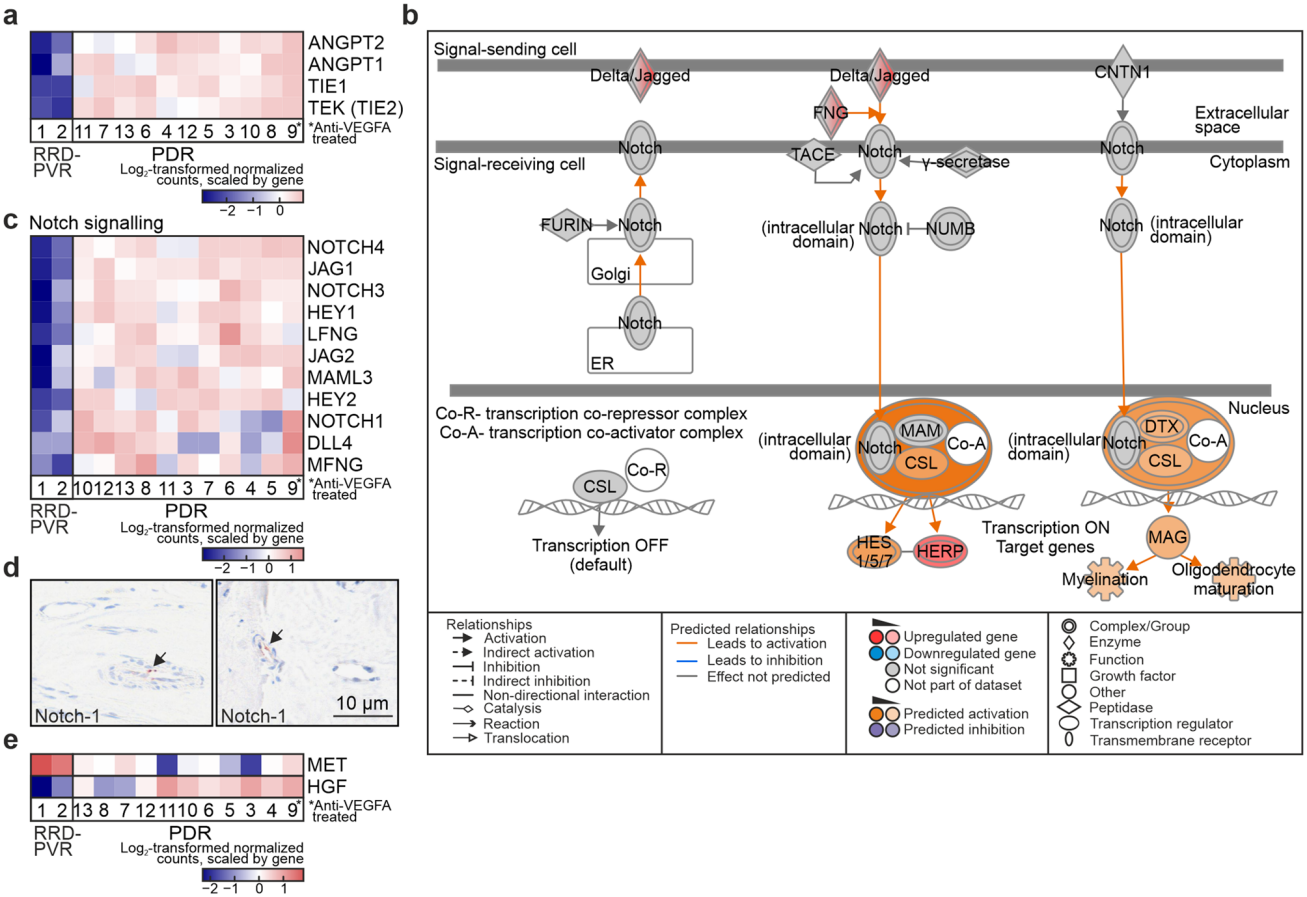
VEGFA-ligand-independent mechanisms are involved in proliferative diabetic retinopathy. (**a**) Heatmap of log_2_-transformed normalized read counts of ANGPT1, ANGPT2, TIE1 and TIE2. (**b**) IPA canonical pathway *Notch signalling*. (**c**) Heatmap of log_2_-transformed normalized read counts of DEGs in *Notch signalling*. (**d**) Representative light micrographs of immunohistochemistry for Notch-1 in PDR fibrovascular tissue. Scale bar, 10 µm. (**e**) Heatmap of log_2_-transformed normalized read counts of MET and HGF. Asterisk in (**a**), (**c**) and (**e**) indicate the anti-VEGFA-treated PDR patient (Patient 9). The pathway in (**b**) was generated through the use of IPA (QIAGEN Inc., https://www.qiagenbioinformatics.com/products/ingenuity-pathway-analysis)^45^.

Not all patients benefit from current anti-VEGFA therapy, suggesting that VEGFA-independent pathways also contribute to PDR^2^. Angiopoietin 1 (ANGPT1) promotes blood vessel maturation and stability via signalling through tyrosine kinase with immunoglobulin and epidermal growth factor homology domains (TIE)-2 receptor^8^. ANGPT2/TIE2-signalling induces vessel destabilization. Both ANGPT1 and ANGPT2 were upregulated in PDR, as were TIE1 and TIE2 (Fig. 4A), suggesting that both vessel-stabilizing and -destabilizing signalling operate in PDR.

ANGPT1/TIE2-signalling can promote other anti-VEGFA therapy escape mechanisms, including delta like ligand 4 (Dll4)/Notch-1 signalling by inducing Wnt/β-catenin pathway^8^. *Notch signalling* and *Wnt/β-catenin–signalling* IPA canonical pathways were predicted to be activated (Fig. 4. B, Supplementary Fig. S2A-B). Notch-1, -3, and -4 were upregulated in PDR, as were Notch ligands Dll4, Jag1 and Jag2 (Fig. 4C). Interestingly, the expression of Notch-1 and Dll4 was higher in the single anti-VEGFA-treated PDR patient, implicating the Notch-1/Dll4 pathway as a possible anti-VEGFA therapy escape mechanism (Fig. 4C). Notch-1 was detected in lumen-lining cells in the PDR tissues (Fig. 4D).

Activin receptor-like kinase 1 (ALK-1, ACVRL1), receptor found on proliferating ECs and involved in TGF-β–mediated neovascularization, was upregulated in PDR (Supplementary Table S3^8^). The hepatocyte growth factor (HGF)/cMet pathway has been suggested as an anti-VEGFA therapy escape mechanism^8,9^. HGF was upregulated in PDR. Though the MET gene was downregulated, within this group its expression was highest in the anti-VEGFA-treated patient (Fig. 4E). Altogether, these results suggest involvement of VEGFA-ligand-independent pathways in PDR, which may work as anti-VEGFA therapy escape mechanisms.

### Genes and processes related to lymphatic development are overrepresented in proliferative diabetic retinopathy

**Figure 5:**
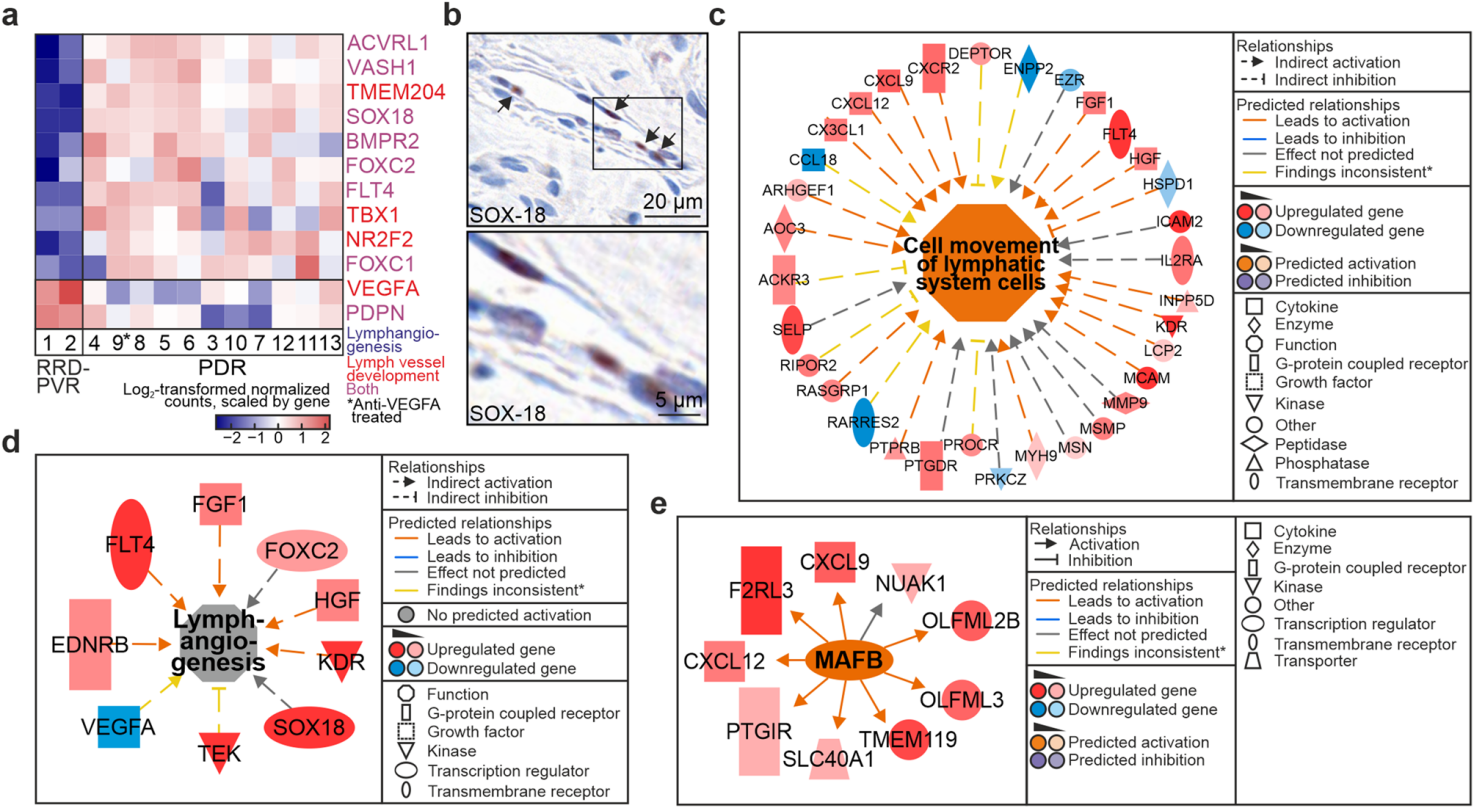
Genes and processes related to lymphatic development are overrepresented in proliferative diabetic retinopathy. (**a**) Heatmap of log_2_-transformed normalized read counts of DEGs in GO-terms *Lymph vessel development* and *Lymphangiogenesis*. (*) Patient treated with intravitreal anti-VEGFA. (**b**) Representative light micrographs of immunohistochemistry of nuclear SOX-18 in PDR fibrovascular tissue. Scale bars, 20 µm (upper), 5 µm (lower). (**c**) Network of DEGs based on which IPA function *Cell movement of lymphatic system cells* was predicted to be activated. (**d**) Network of DEGs based on which IPA function *Lymphangiogenesis* was significantly affected, though not predicted to be activated. (**e**) IPA Upstream Regulator MAFB was predicted to be activated. (*) Yellow arrows indicate findings inconsistent with the state of downstream molecule. The networks in (**c**), (**d**) and (**e**) were generated through the use of IPA (QIAGEN Inc., https://www.qiagenbioinformatics.com/products/ingenuity-pathway-analysis)^45^.

In addition to angiogenesis, formation of lymphatic-like structures has been implicated in PDR^10^. GO-terms *Lymph vessel development* and *Lymphangiogenesis* were significantly enriched based on DEGs upregulated in PDR (Fig. 5A, Supplementary Table S5A). Ten of 23 genes in *Lymph vessel development* and seven of 15 in *Lymphangiogenesis* were upregulated. The main lymphangiogenic growth factors VEGFC and -D were not DEGs, but their receptor VEGFR3 (FLT4) was upregulated, as was TBX1, which can activate VEGFR3 transcription in ECs (Fig. 5A^11^). The poorly studied hypoxia-inducible transmembrane protein 204 (TMEM204), which can interact with VEGFR2 and -3 to regulate lymphatic vessel development^12^, was also upregulated in PDR (Fig. 5A).

VEGFR3 is common in lymphatic endothelial cell (LEC) precursors, as are SOX18 and NR2F2. NR2F2 and SOX18 induce PROX1 expression in LEC specification^13^. Both SOX18 and NR2F2 were upregulated in PDR (Fig. 5A). Nuclear SOX18 was found in lumen-lining cells in PDR tissues (Fig. 5B). NR2F2 and FOXC2 are expressed in established lymphatic vessels^13^. FOXC2 and GATA2, a transcription factor participating in PROX1, FOXC2 and NFATC1 regulation in lymphatic valve development, were upregulated in PDR (Supplementary Table S3;^14^). *Cell movement of lymphatic system cells*, *Migration of lymphatic system cells, Lymphangiogenesis* and *Proliferation of lymphatic endothelial cells* were significantly increased functions in IPA downstream effects analysis (Fig. 5C-D; Supplementary Fig. S3A-B).

MAFB is expressed in LECs, upregulated by VEGFC/VEGFR3-signalling and involved in lymphatic differentiation through regulation of VEGFR-3, LYVE-1, podoplanin, (PDPN), Prox-1 and Klf4 expression^15^. Though not a DEG, MAFB was predicted an activated UR (Fig. 5E). PDPN was downregulated in PDR and LYVE1 and PROX1 were not DEGs, but all three showed interpatient variability (Supplementary Table S3). Variability was also observed by immunohistochemistry of PDR tissues^10,16^. Altogether, these results suggest that LEC programs contribute to PDR pathological neovascularization.

### Inflammatory and wound healing responses are involved in both PDR and RRD-PVR

**Figure 6:**
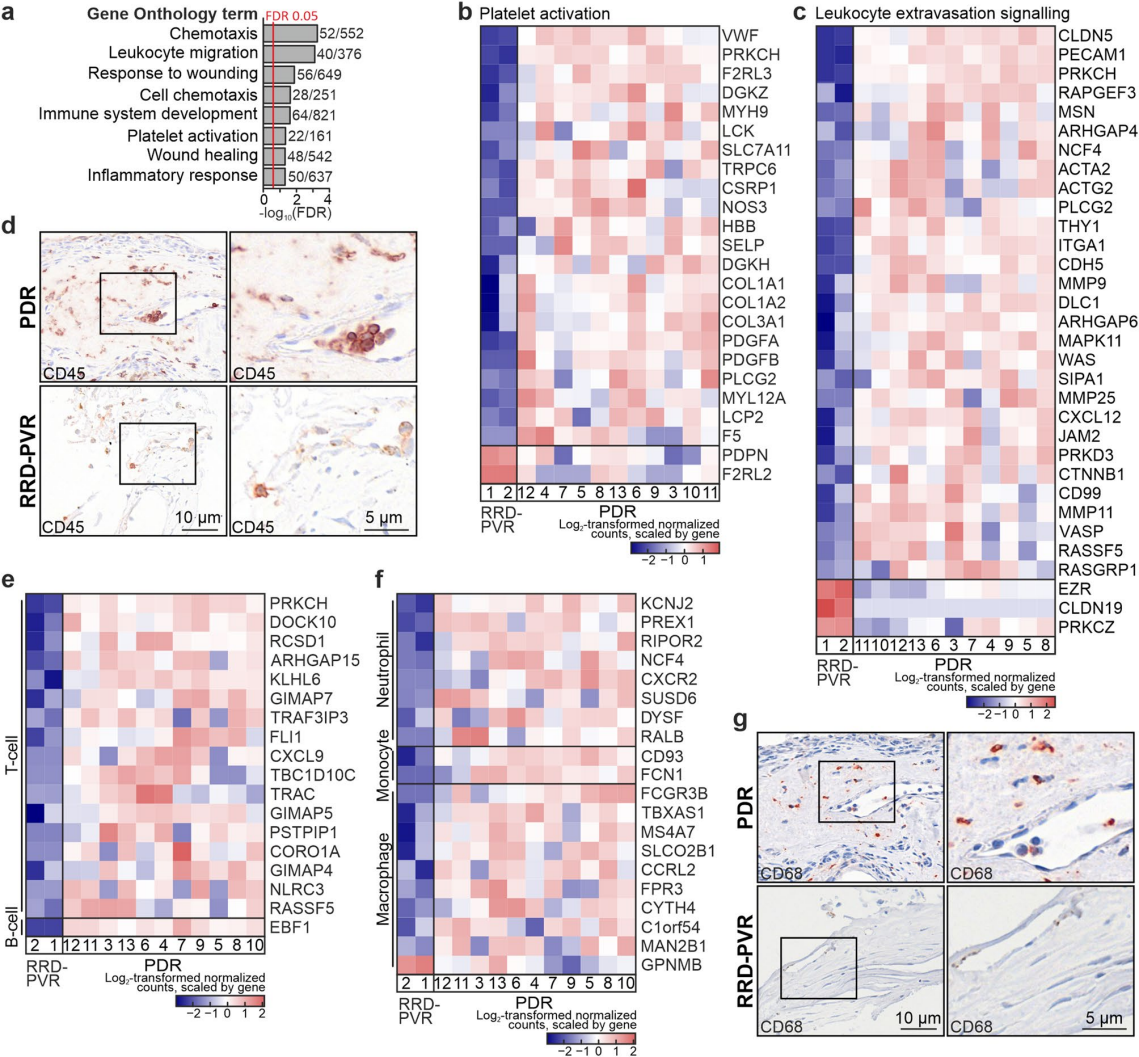
Inflammation and wound healing are involved in PDR and RRD-PVR. (**a**) Bar chart showing GO-terms related to inflammation and wound healing, based on the DEGs upregulated in PDR. Numbers beside bars indicate number of DEGs in GO-term versus the total number of genes in the GO-term. (**b**) Heatmap of log_2_-transformed normalized read counts of DEGs in GO-term *Platelet activation*. (**c**) Heatmap of log_2_-transformed normalized read counts of DEGs in IPA canonical pathway *Leukocyte extravasation signalling.* (**d**) Representative light micrographs of immunohistochemistry for CD45 in excised PDR and RRD-PVR tissue. (**e–f**) Heatmap of log_2_-transformed normalized read counts of DEGs in T-cell and B-cell signatures (**e**), and in macrophage, monocyte, and neutrophil cell signatures (**f**). (**g**) Representative light micrographs of immunohistochemistry of CD68 in PDR and RRD-PVR tissue. Scale bars, 10 µm (left), 5 µm (magnified insets).

Inflammation and wound healing are relevant to PDR and RRD-PVR^2,5^. GO-terms *Chemotaxis, Leukocyte migration, Response to wounding, Immune system development, Inflammatory response, Coagulation* and *Platelet activation,* as well as KEGG pathways *Platelet activation* and *Leukocyte transendothelial migration* were overrepresented based on DEGs upregulated in PDR (Fig. 6A, Supplementary Table S5D). Of 161 genes in GO-term *Platelet activation,* two genes, also expressed in RPE, were upregulated in RRD-PVR, while 22 were upregulated in PDR, consistent with vascular leakage and haemorrhage only in PDR (Fig. 6B). Of 649 genes in *Response to wounding,* 56 were upregulated in PDR, while 26 were upregulated in RRD-PVR (Supplementary Fig. S4A), consistent with wound healing in both diseases.

Of 637 genes in GO-term *Inflammatory response*, 50 were upregulated in PDR, and 16 upregulated in RRD-PVR (Supplementary Fig. S4B). IPA canonical pathway *Leukocyte extravasation signaling* was activated. Of 193 genes in this pathway, 29 were upregulated and three downregulated in PDR (Fig. 6.C). The functions *Leukocyte migration, Cell movement of mononuclear leukocytes, Migration of mononuclear leukocytes, Binding of leukocytes* and *Cell movement of lymphocytes* were increased in IPA (Supplementary Fig. S4C-E, Supplementary Table S6D). Presence of CD45-positive leukocytes was confirmed in PDR and RRD-PVR tissues (Fig. 6D). The inflammatory cytokine CCL5 scored as an activated UR (Supplementary Table S6B). When analyzing the expression of a set of immune cell signature genes^17^, 17 of 85 T-cell signature genes and one of 37 B-cell signature genes were upregulated in PDR (Fig. 6E). NK-cell and plasma cell signature genes were not DEGs. Eight of 47 neutrophil, two of 37 monocyte and nine of 78 macrophage signature genes were also upregulated in PDR (Fig. 6F). One macrophage signature gene (GPNMB), also expressed in RPE^18^, was upregulated in RRD-PVR. CD68-positive macrophages were found in PDR tissues, but not in the two RRD-PVR tissues (Fig. 6G).

### Broader retinal functions and retinal pigment epithelium gene signature are enriched in rhegmatogenous retinal detachment with proliferative vitreoretinopathy

Despite similarities with PDR, RRD-PVR presents distinct features. Based on DEGs upregulated in RRD-PVR, 208 BPs, 23 CCs and 15 MFs and one KEGG pathway were significant in GOEA (Supplementary Fig. S5A, Supplementary Table S7A-D). BPs related to retinoid metabolism, eye morphogenesis, and visual perception, and CCs related to extracellular exosomes, ribosomes, and pigment granules, were enriched (Supplementary Fig. S5A, Supplementary Table S7A-B).

Since RPE cells are known to contribute to RRD-PVR pathogenesis^5^, we investigated the expression of a set of RPE cell signature genes^18^. Of these genes, 31.8% were upregulated in RRD-PVR, while 1.3% were downregulated (Supplementary Fig. S5B), suggesting presence of RPE cells in RRD-PVR tissues. Marker genes of other retinal cell types were also upregulated in RRD-PVR, such as CLU, GLUL and RLBP1 (Müller cells and astrocytes^19,20^), TRPM1 and OTX2 (bipolar cells^19,21^), TMEM119 (microglia^22^), SNCG (retinal ganglion cells^19^), and GNGT1 (photoreceptor cells^23^).

### The epithelial-to-mesenchymal transition (EMT) is overrepresented in proliferative diabetic retinopathy

**Figure 7:**
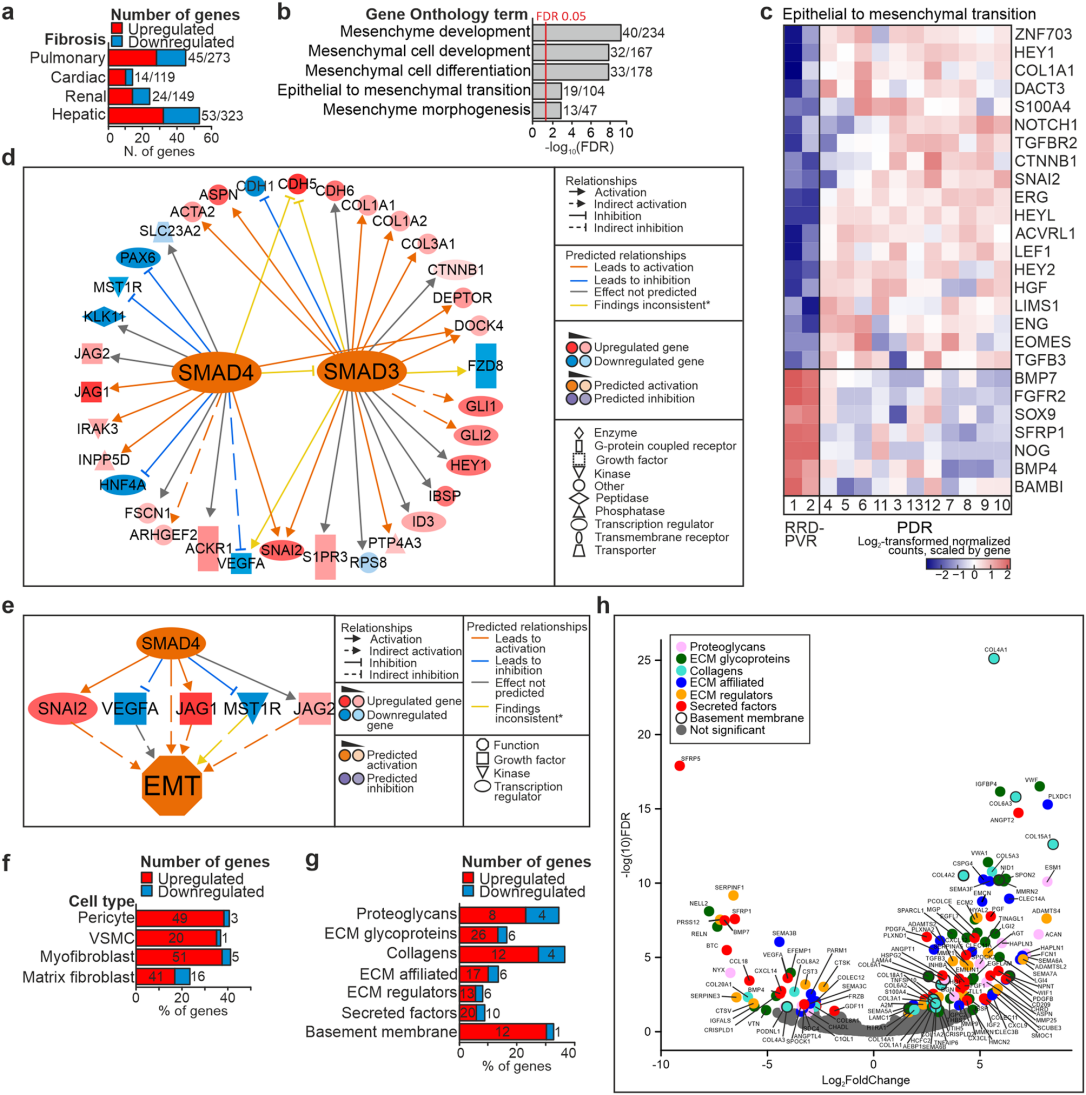
Epithelial to mesenchymal transition and the extracellular matrix changes are involved in PDR and RRD-PVR. (**a**) Stacked bar chart summarizing the number of DEGs (upregulated, red; downregulated, azure) among the pulmonary, cardiac, renal and hepatic fibrosis signature genes^24^. Numbers beside bars indicate number of DEGs in the signature versus the total number of genes in the signature. (**b**) Bar chart showing GO-terms related to epithelial to mesenchymal transition (EMT) and mesenchyme development, significantly enriched based on DEGs upregulated in PDR. Numbers beside bars indicate the number of significant genes in GO-term versus the total number of genes in GO-term. (**c**) Heatmap of log_2_-transformed normalized read counts of DEGs in the GO-term *Epithelial to mesenchymal transition*. (**d**) The function of SMAD3 and SMAD4 was predicted to be activated. (**e**) SMAD4 was found within the regulator effect network with targets SNAI2, VEGFA, JAG1, JAG2 and MST1R, and the activated effect *Epithelial-mesenchymal transition (EMT).* (*) Yellow arrows indicate findings inconsistent with the state of downstream molecule. (**f**) Stacked bar chart summarizing the percentage of DEGs (upregulated, red; downregulated, azure) among “vascular smooth muscle cell”, “pericyte”, “myofibroblast” and “matrix fibroblast” cell signature genes^28^. Numbers in brackets beside bars indicate the total number of genes in each signature (**g**) Stacked bar charts summarizing the percentage of DEGs among the matrisome signature genes^30^. (**h**) Volcano plot depicting the differential expression of matrisome genes^30^. DEGs are color-coded as indicated, number of genes indicated in or adjacent to bars. The networks in (**d**) and (**e**) were generated through the use of IPA (QIAGEN Inc., https://www.qiagenbioinformatics.com/products/ingenuity-pathway-analysis)^45^.

In PDR and RRD-PVR, fibrotic responses occur together with inflammation. For a broader view of the fibrotic signatures in these diseases, we investigated the presence of DEGs among hepatic, renal, cardiac or pulmonary fibrosis signature genes catalogued in FibroAtlas^24^. DEGs were present in all categories, and included both upregulated and downregulated genes, suggesting diverse fibrotic mechanisms in both diseases (Fig. 7A).

The myofibroblasts, major drivers of fibrosis, can originate from resident fibroblasts, circulating fibrocytes, or through transdifferentiation of epithelial or endothelial cells^25,26^. BPs *Mesenchyme development, Mesenchymal cell development, Mesenchymal cell differentiation, Mesenchyme morphogenesis* and *Epithelial-to-mesenchymal transition* were enriched based on DEGs upregulated in PDR (Fig. 7B; Supplementary Table S5A). Of 104 genes in *Epithelial-to-mesenchymal transition*, 19 were upregulated, and seven downregulated in PDR (Fig. 7.C). Several genes upregulated in RRD-PVR were expressed in RPE cells^18^ which may contribute to membrane formation via EMT^27^. Consistently, the BP *Mesenchymal cell differentiation* was also enriched based on DEGs upregulated in RRD-PVR (Supplementary Table S7A).

IPA canonical pathways *Regulation of the EMT in development* and *TGF-β signaling pathway* as well as the function *EMT* were activated (Supplementary Fig. S6A-B, Supplementary Table S6A and D). In significantly altered canonical pathway *Regulation of the EMT,* the function *EMT* was predicted to be activated through TGF-β signaling and SMAD2, -3 and -4 activation (Supplementary Fig. S6C). TGF-β1, SMAD3 and SMAD4 scored as activated URs (Fig. 7D, Supplementary Fig. S6D). SMAD4 was also found within the regulator effect network with targets SNAI2, VEGFA, JAG1, JAG2 and MST1R, and activated effect *EMT* (Fig. 7E). SMAD2 and -3 are downstream of TGF-β, while SMAD1, -5 and -8 are downstream of BMPs. BMP4 and SNAI1 scored as activated URs. These results suggest that fibrosis in PDR can occur through regulation of EMT.

To gain further insight into the cell populations likely involved in the PDR and RRD-PVR fibrotic processes, we analyzed the expression of a set of “pericyte”, “vascular smooth muscle cell”, “myofibroblast” and “matrix fibroblast” signature genes retrieved from the LungGENS database^28^. DEGs were present in all categories (Fig. 7F, Supplementary Fig. S7A-D). Consistent with absence of vasculature in RRD-PVR, only one and three “vascular smooth muscle cell” and “pericyte” signature genes, respectively, were upregulated in RRD-PVR. Notably, only 9% of the DEGs among the “myofibroblast” signature genes were upregulated in RRD-PVR, compared to 28% in the “matrix fibroblast” signature. These results suggest that cells with “matrix fibroblast” signature are more likely than cells with “myofibroblast” signature to contribute to RRD-PVR fibrotic processes.

### Several extracellular matrix -related genes and processes enriched in proliferative diabetic retinopathy

Fibrosis involves ECM deposition, and ECM composition can affect treatment responses^29^. GO-terms *Extracellular matrix* and *ECM structural constituent* were enriched based on DEGs upregulated in PDR, as was KEGG pathway *ECM-receptor interaction* (Supplementary Table S5). To investigate ECM composition differences, we evaluated the differential expression of a set of 1013 matrisome genes^30^. Of these, 132 were DEGs, including 13 basement membrane genes, 16 collagen genes, 19 ECM regulator genes, 12 proteoglycan genes, 31 ECM glycoprotein genes, 23 ECM affiliated genes and 30 secreted factor genes (Fig. 7G-H), indicating differences in ECM composition between PDR and RRD-PVR.

## Discussion

Global mRNA expression in PDR and RRD-PVR tissues has not been investigated by sequencing, though DNA microarray analysis of PDR tissues has revealed gene expression differences between PDR tissues and normal retina^3,31^. Lack of comprehensive knowledge on the mechanisms and factors involved in formation of these membranes has hindered the development of novel treatments. In this study, we compared gene expression of PDR and RRD-PVR tissues to find mechanisms specific to these diseases.

As expected, multiple angiogenic pathways were overrepresented in PDR. Rather than low absolute expression in PDR tissues, the observed downregulation of VEGFA in PDR is likely to reflect prominent VEGFA level in RRD-PVR tissues, as VEGFA is involved in PVR pathogenesis^32^. Other angiogenic factors besides VEGFA have been detected in PDR vitreous, such as Ang-2 and MMP9^2,10,33^. VEGFA-ligand-independent pro-angiogenic pathways may explain why many patients experience progression of neovascularization even after initial success with photocoagulation and/or anti-VEGFA therapies^2,34^. PlGF-VEGFR1, ANGPT1/2-TIE2, Dll4/Notch-1, HGF/cMet and ALK-1 related mechanisms were overrepresented in PDR, highlighting the importance of combination treatments. PlGF/VEGFA-VEGFR1 signalling has been suggested as a pathogenesis mechanism in diabetic retinopathy^35^. Novel therapeutics are under study, including anti-Ang-2/anti-VEGFA bispecific antibody Faricimab (RG7716, Roche)^36^, recently approved in the US for treatment of DME and wet age-related macular degeneration (AMD)^37^. ALK-1 involvement in angiogenesis is not fully understood, but combined targeting of VEGFR and ALK-1 has been suggested for inhibition of anti-VEGFA-resistant angiogenesis in cancer^8^.

Consistent with our previous results^10,16^, we identified overrepresentation of lymphatic development-related GO-terms and pathways. Our results also show upregulation of VEGFR3, TBX1, SOX18 and NR2F2 in PDR, consistent with LEC programming. PROX1 and LYVE1 were not differentially expressed, but had variable expression among PDR patients, consistent with our previous findings^10,16^. Lymphatic marker podoplanin is not expressed in endothelium in PDR tissues^16^, and is also expressed in RPE cells^18,38^. Therefore, the upregulation of podoplanin in RRD-PVR likely reflects abundance of RPE cells in RRD-PVR tissues.

Inflammation, fibrosis, and wound healing are relevant to both RRD-PVR and PDR^2,6,29^. Myofibroblasts that originate from endothelial-to-mesenchymal transition (EndMT) are suggested to contribute to fibrosis in PDR fibrovascular membrane formation, and factors that regulate EMT can also regulate EndMT^26,39^. Therefore, the observed overrepresentation of EMT processes in PDR may result from the presence of vasculature. Mesenchymal cell differentiation was also implicated in RRD-PVR, consistent with fibrotic tissue formation via EMT from RPE cells^6^. Fibrosis also involves ECM deposition, and we identified differential matrisome gene expression between PDR and RRD-PVR. In the future, in-depth analysis of the ECM composition can help define the complex cell-ECM interactions and the physical microenvironment within fibrotic tissue formation.

Our current study is restricted to analyses of mRNA expression. Previously, we compared the proteomes of PDR and DR vitreous^40^ and found GO-terms related to inflammation, immune response, wound healing, and coagulation significantly enriched in PDR. This is consistent with the enriched GO-terms and transcriptional pathways found here in the PDR tissues. As the proteome of a tissue may exhibit variability not detectable in its transcriptome, comparative validation of both the proteome and transcriptome of the pathological PDR tissues remains of future interest.

In absence of healthy control tissue, the avascular RRD-PVR tissues enable us to explore the PDR transcriptome, especially with regards to the PDR vascular aspects. Expression patterns related to inflammation, fibrosis, and wound healing found in our data instead reflect the specific differences between these mechanisms central to both RRD-PVR and PDR. Nevertheless, this comparison can serve as a basis for future individual investigations of the complex fibrotic/fibrovascular and inflammatory mechanisms behind the pathological tissue formation in these two sight-threatening vitreoretinal eye diseases.

Bulk RNA sequencing provides tissue-level gene expression data. Approaches such as single-cell RNA sequencing and spatial transcriptomics will be instrumental to define the tissue heterogeneity and cell-specific expression patterns in fibrovascular PDR formation and avascular RRD-PVR progression.

The small cohort size is another limitation in our current study. For instance, the cohort included only one anti-VEGFA (Ranibizumab) treated patient, ruling out statistical analyses related to this treatment. This patient showed, however, an interesting expression profile suggestive of putative therapy escape mechanisms and further studies will be of interest to investigate the impact of anti-VEGFA and other treatments, e.g. statins, in larger cohorts.

In conclusion, our findings on fibrotic, fibrovascular and inflammatory changes and potential therapy escape mechanisms open new avenues for the study of PDR and RRD-PVR progression and for the development of novel therapies. Further investigation of these processes may also help to understand other devastating posterior-segment vitreoretinal diseases where similar pathological mechanisms are involved, such as wet-AMD and retinopathy of prematurity.

## Methods

### Approval and surgery

Patients were enrolled in the tertiary care ophthalmology clinic, unit of vitreoretinal diseases, at Helsinki University Hospital (HUH) Eye Clinic in 2010–2019. Signed informed consent was obtained from each patient. The study was approved by the Institutional Review Board and Ethical committee of HUH and conducted according to the Declaration of Helsinki.

Transconjunctival microincision vitreoretinal surgery by 23- or 20-gauge three-port pars plana vitrectomy was performed as previously described^10^. The indications for vitrectomy were active PDR, with or without TRD threatening or involving the macula, or severe non-clearing VH. For our bulk RNA cohort, 11 PDR membranes were excised from 11 vitrectomized PDR eyes of 11 patients using segmentation and delamination, cut when needed with microscissors, and removed from the vitreous cavity with intraocular end-gripping microforceps (MaxGrip Alcon Laboratories). The PDR patients had type 1 DM. Of the 11 PDR patients, one received pre-operative intravitreal VEGFA and all except one received previous photocoagulation. Summary baseline characteristics of patients can be found in Supplementary Table 1, and individual characteristics in Supplementary Table 2.

For bulk RNA sequencing, excised tissues were immersed in RNA stabilization reagent (RNA later, Qiagen) and stored at − 80 °C until processing. Tissue samples utilized for immunohistochemistry were obtained and processed as previously described^16^.

### RNA extraction and bulk sequencing

The RNeasy Fibrous Tissue Mini kit (Qiagen) was utilized for total RNA extraction, using ceramic beads (MP Biomedicals) and TissueLyser-II for homogenization of tissue. Preparation of total RNA, washes and elution were performed according to kit protocol. The Agilent Bioanalyzer RNA-nano chip (Agilent) was used for evaluation of RNA integrity, and Qubit RNA kit (Life Technologies) for RNA quantification. Extracted RNA was stored at − 80 °c until library preparation, evaluation and sequencing, which were performed as previously described^10^.

### Antibodies

Mouse monoclonal antibodies against CD31 (1:100; M0823; Dako), Notch-1 (1:50; sc-376403; Santa Cruz; CA, USA), SOX18 (1:100; sc-166025; Santa Cruz,) and CD45 (1:100; M070129-2; Dako) and rabbit polyclonal antibodies against CD68 (HPA_048982; 1:3000; Sigma) were used.

### Immunohistochemistry

Immunohistochemistry (IHC) was performed as previously described^10^. Heat-induced antigen retrieval (0.1 M sodium citrate, 15 min) was used. Images were taken using 3DHISTECH Pannoramic 250 FLASH II digital slide scanner.

### Data analysis

Differential expression analysis was performed using HiSeq 2000 platform (Illumina), Lexogen Quantseq 2.2.3 FWD pipeline on BlueBee genomics cloud-based analysis platform. Hierarchical clustering was performed based on the differentially expressed genes (DEGs, with false discovery rate (FDR) < 0.05), and principal component (PC) analysis on the 500 most variable genes, using R Studio (R Core Team, 2020)^41^. Gene ontology (GO)-term and Kyoto Encyclopaedia of Genes and Genomes (KEGG) enrichment analysis^42^ was performed with The Database for Annotation, Visualization and Integrated Discovery (DAVID) Bioinformatics Resources 6.8^43,44^, and R Studio (R Core Team, 2020)^41^. *P* values were adjusted using FDR with a 0.05 cut-off. Pathway analysis was generated through the use of Ingenuity Pathway Analysis (IPA, https://www.qiagenbioinformatics.com/products/ingenuity-pathway-analysis, QIAGEN Inc.^45^) on the DEGs.

## Supplementary Information

Supplementary Information 1.

Supplementary Information 2.

Supplementary Information 3.

Supplementary Information 4.

Supplementary Information 5.

Supplementary Information 6.

Supplementary Information 7.

Supplementary Information 8.
