# Supplementary material for: Proliferative diabetic retinopathy transcriptomes reveal angiogenesis, anti-angiogenic therapy escape mechanisms, fibrosis and lymphatic involvement
Source: Sci Rep. 2021 Sep 22;11:18810. doi: 10.1038/s41598-021-97970-5 (PMC8458546; doi:10.1038/s41598-021-97970-5)
Supplement: Supplementary file 1 — Supplementary Information 1. [file 41598_2021_97970_MOESM1_ESM.pdf]

Supplementary Material

Proliferative diabetic retinopathy transcriptomes reveal angiogenesis, anti-angiogenic therapy escape mechanisms, fibrosis and lymphatic involvement

Ani Korhonen, Erika Gucciardo, Kaisa Lehti and Sirpa Loukovaara

Table of contents

Supplementary Figures

Supplementary Figure Legends

Supplementary Table Legends

References

a

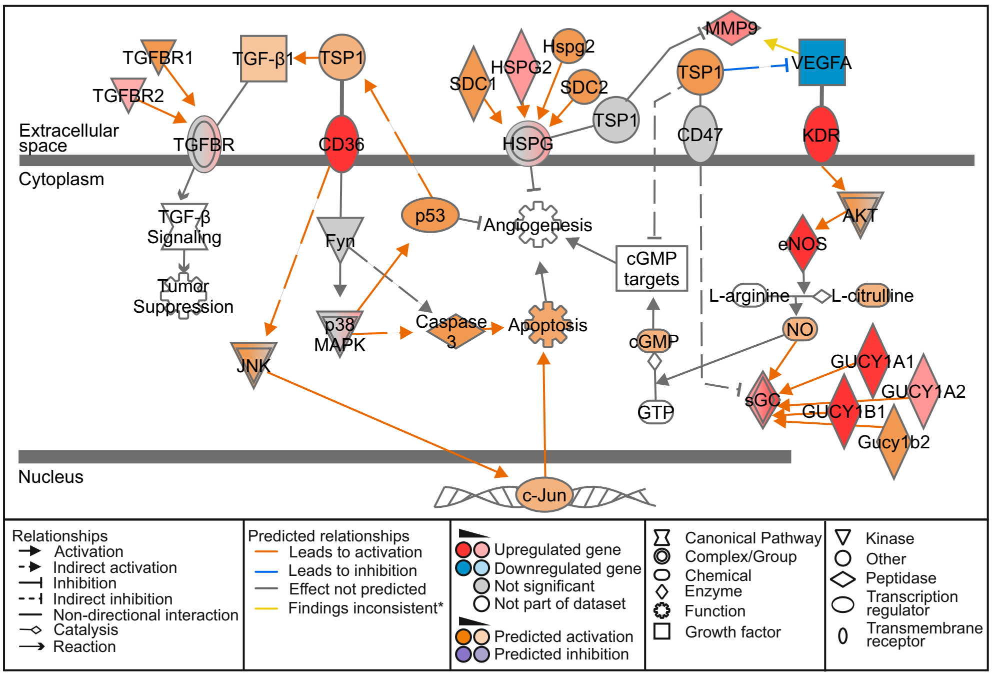

b

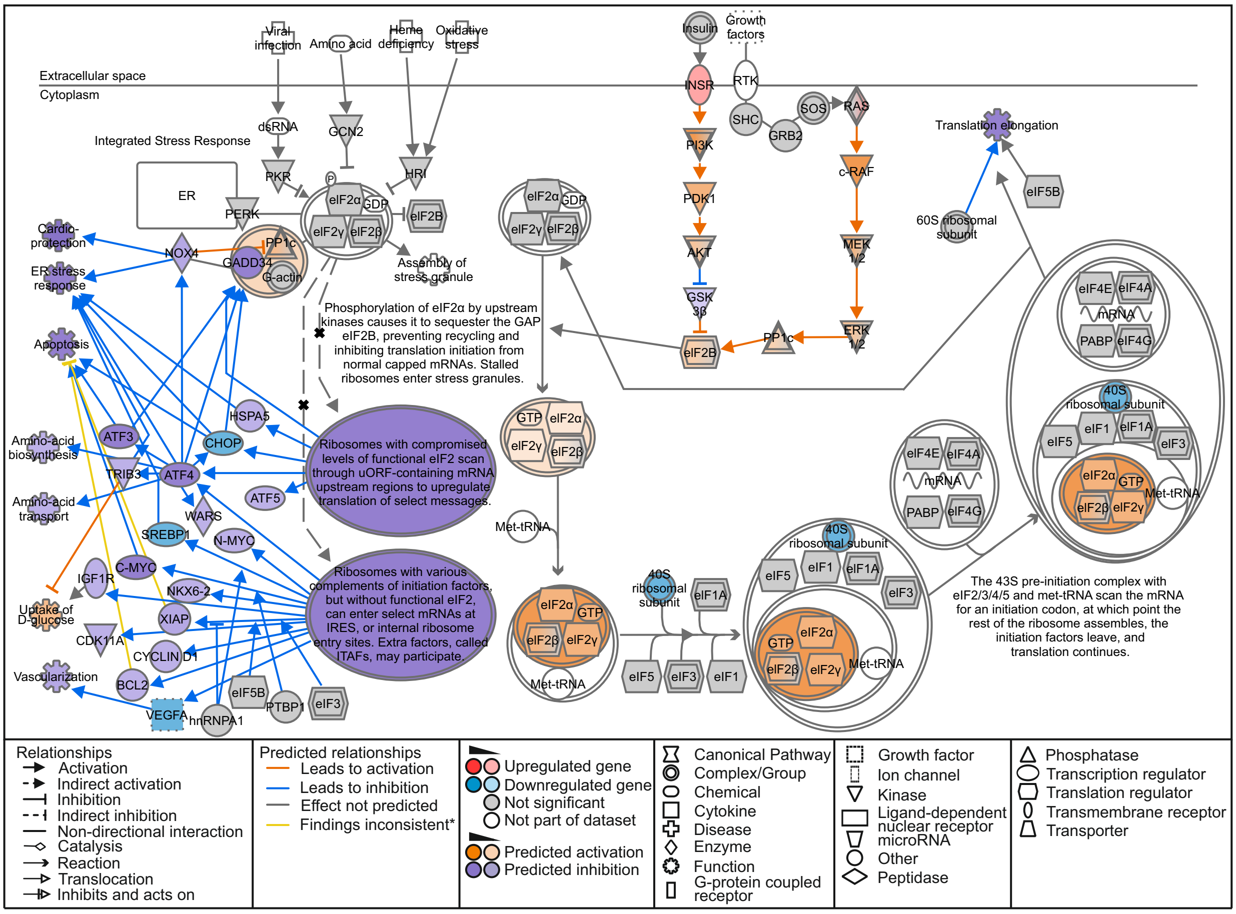

c

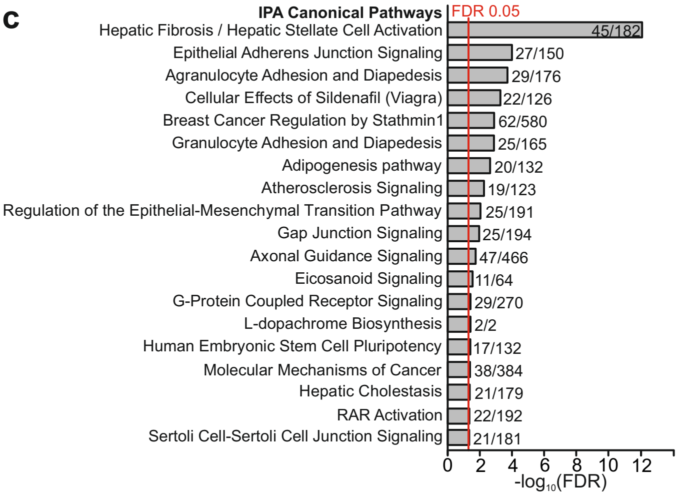

Supplementary Figure S1

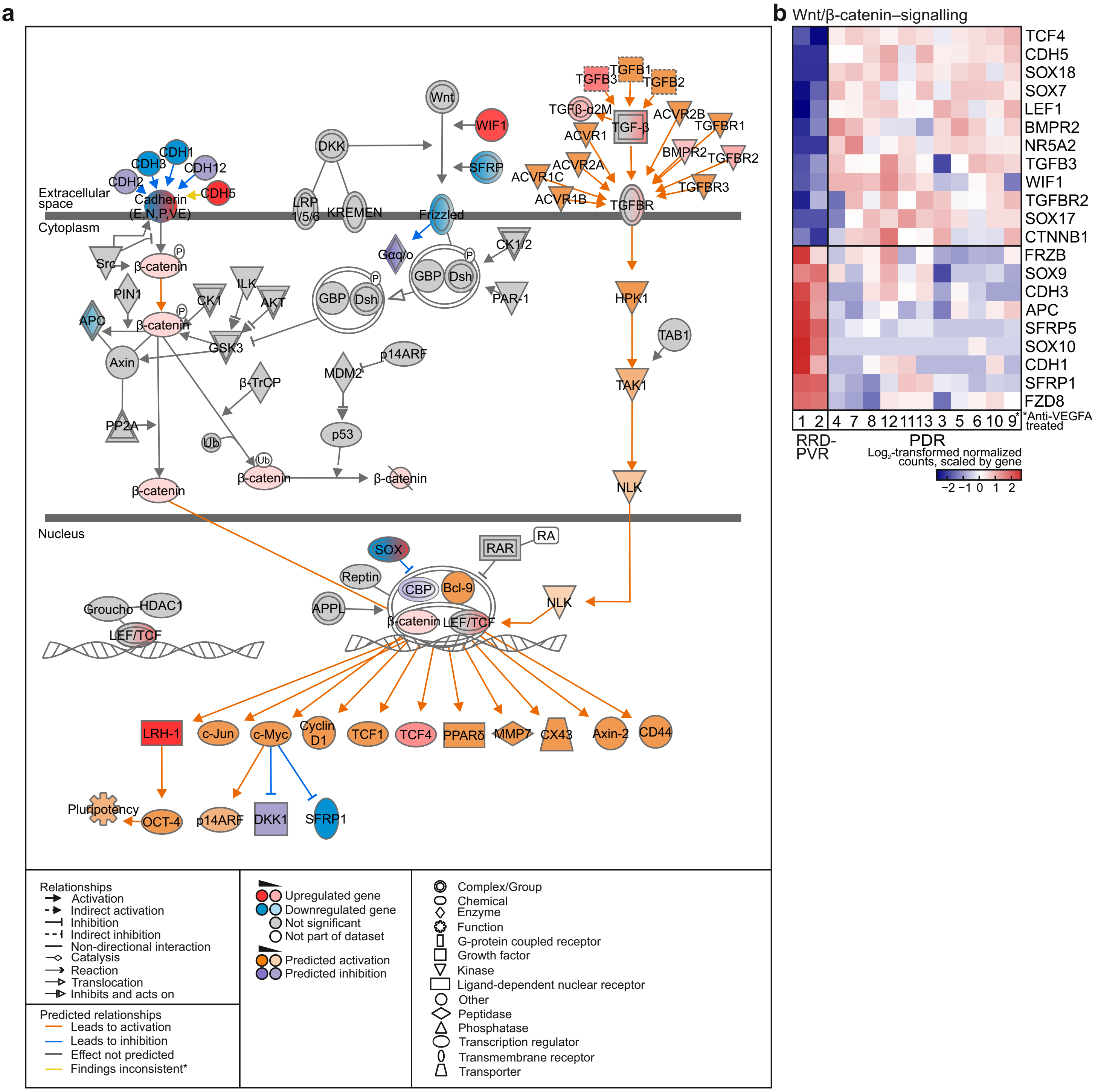

Supplementary Figure S2

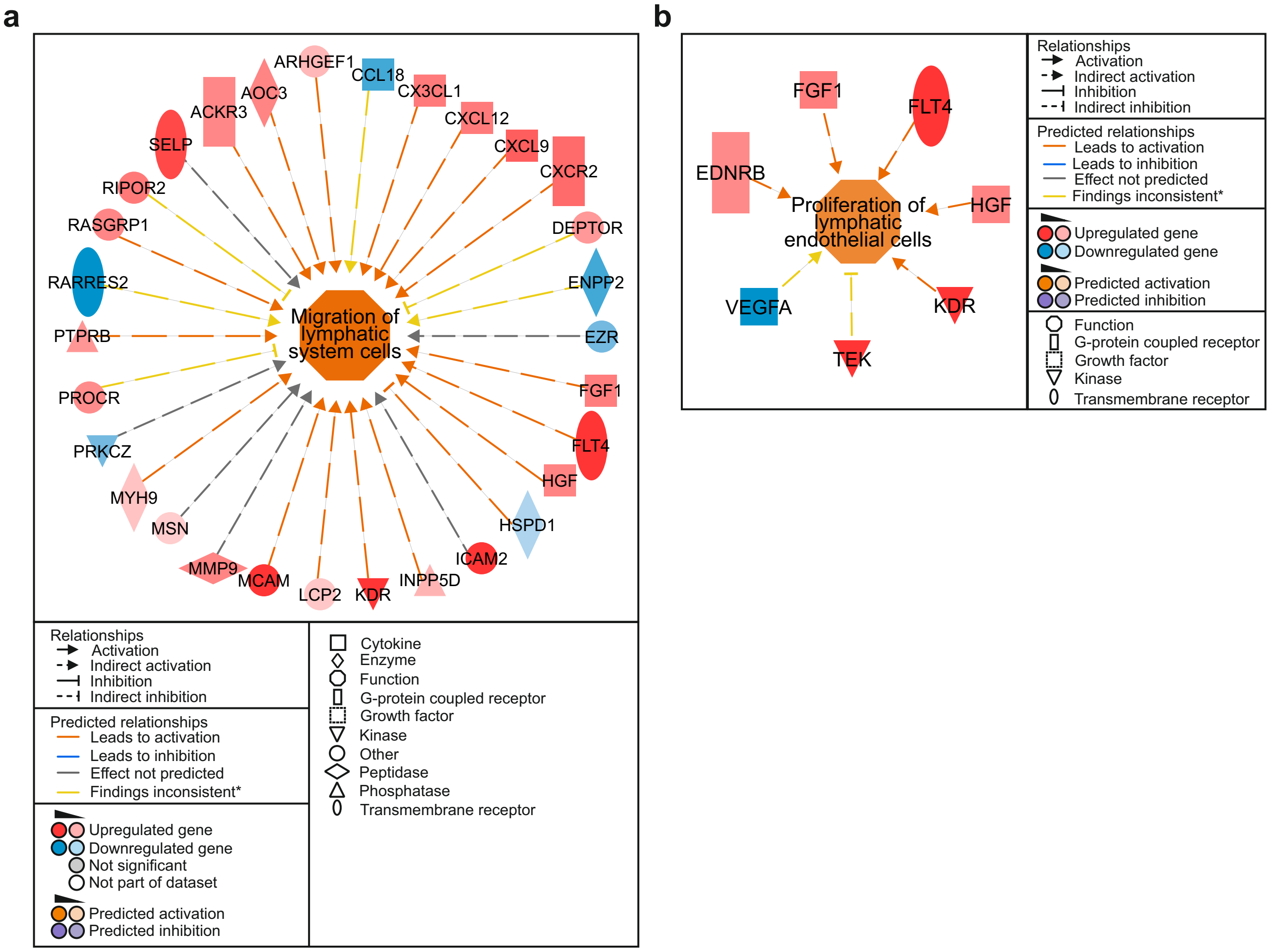

Supplementary Figure S3

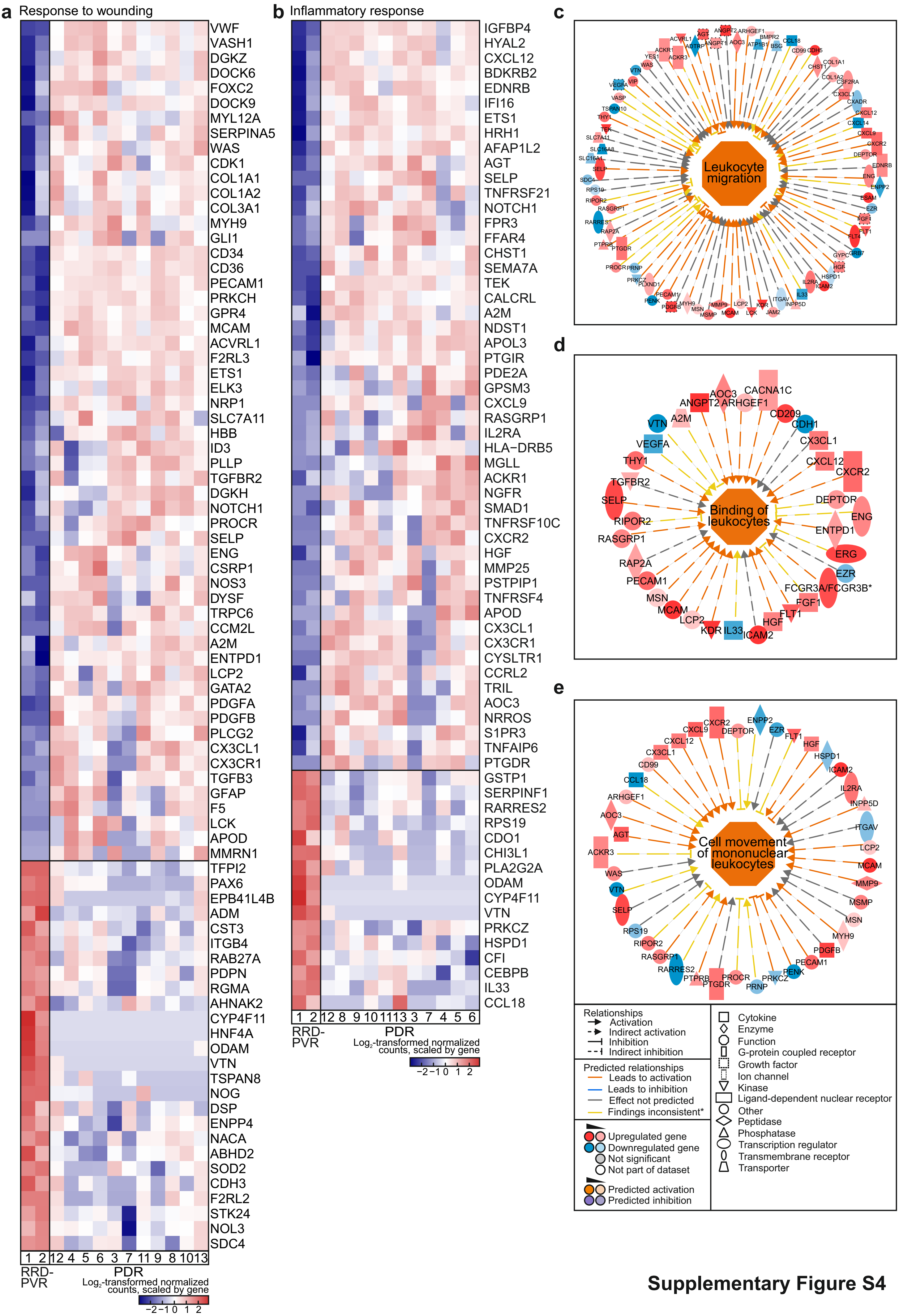

Supplementary Figure S4

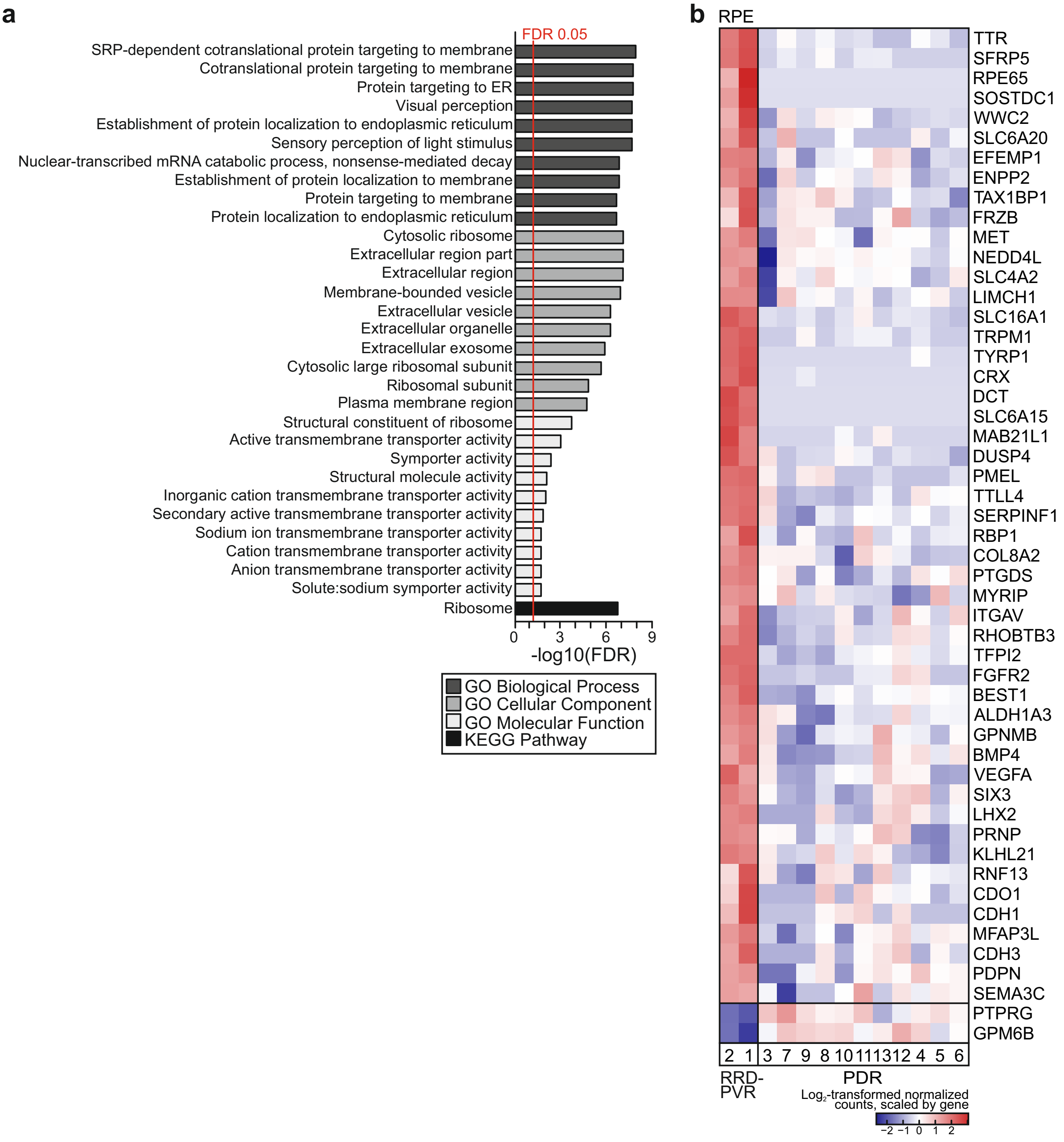

Supplementary Figure S5

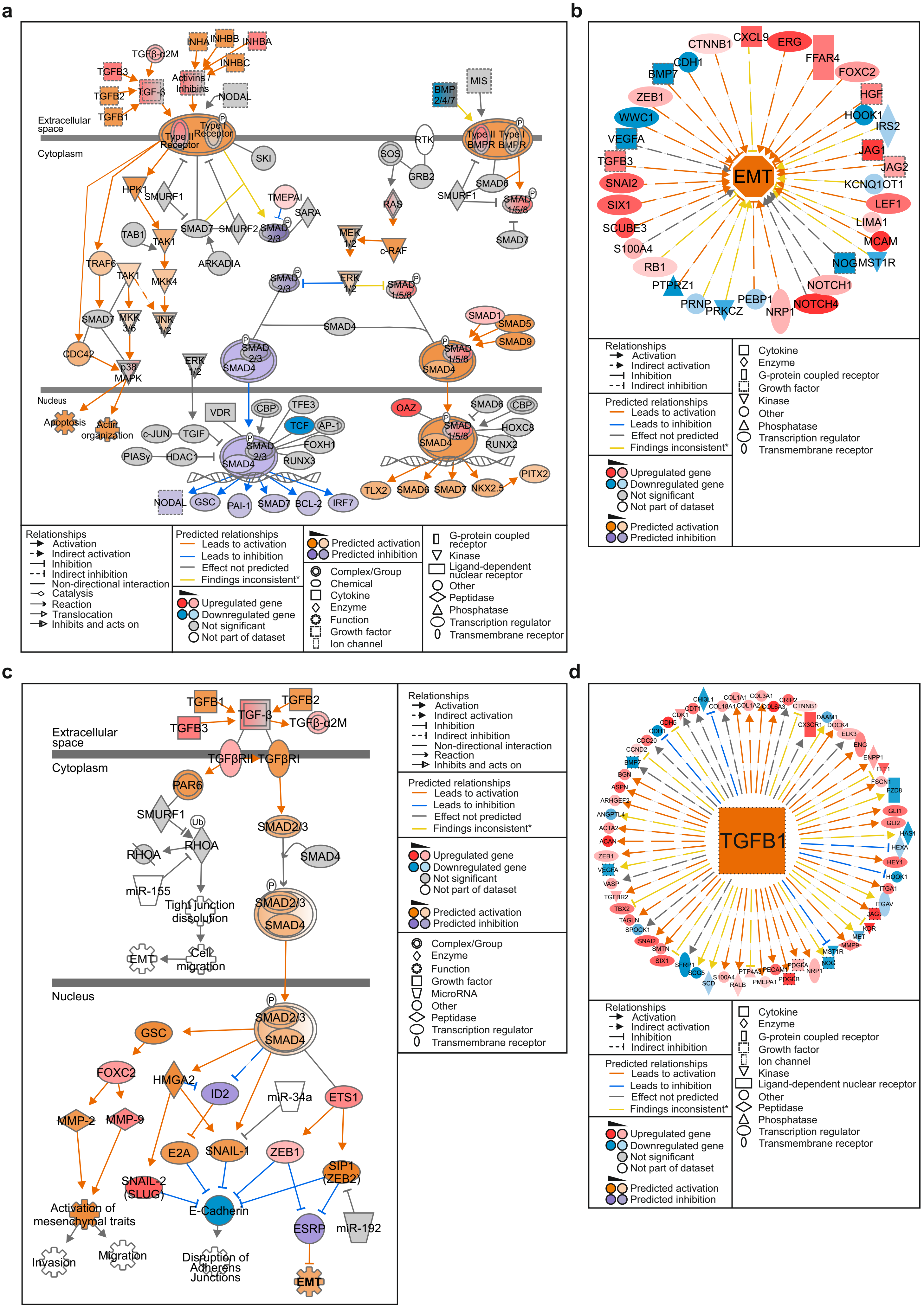

Supplementary Figure S6

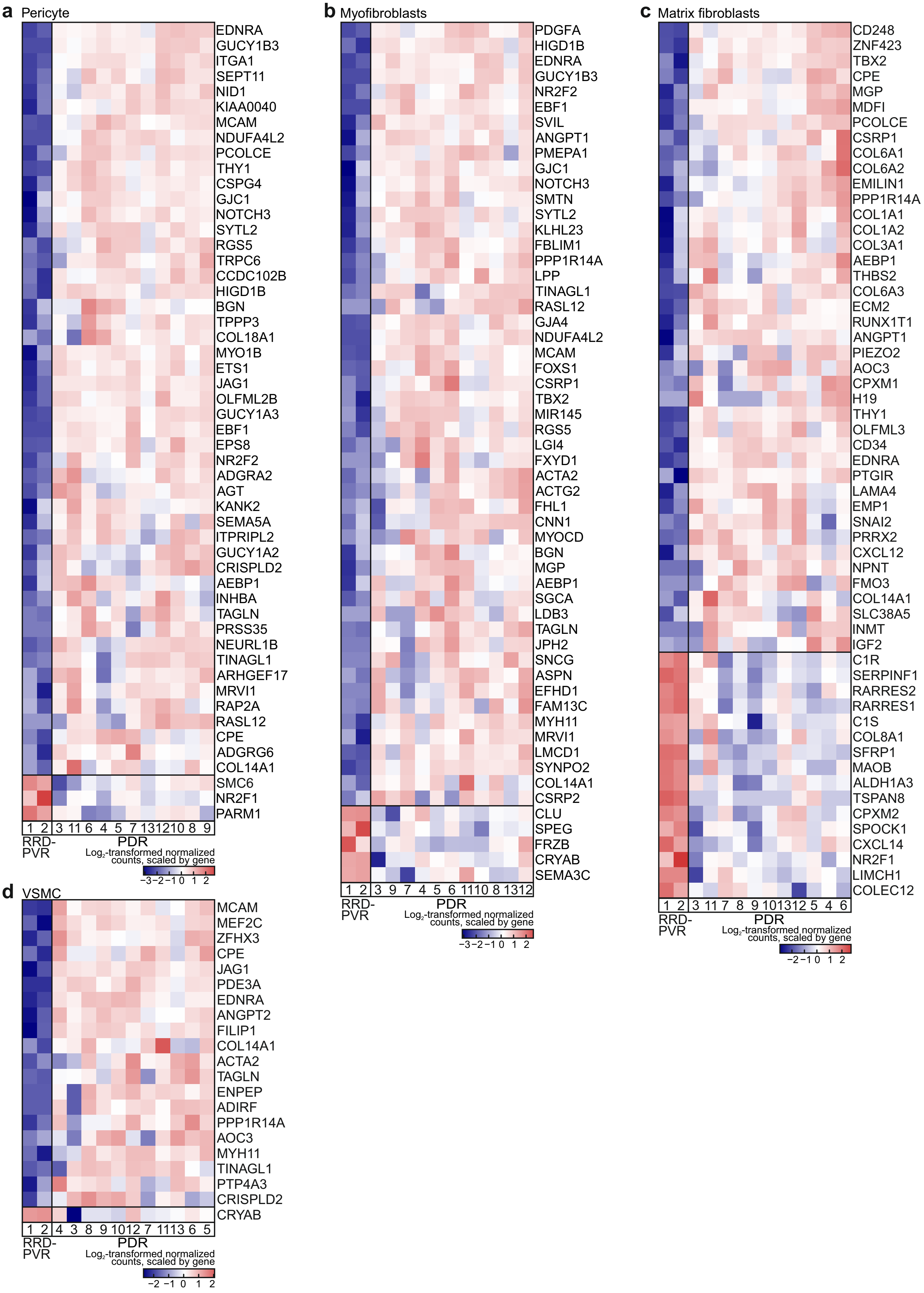

Supplementary Figure S7

Supplementary Figure Legends

Supplementary Figure S1. IPA analyses. (**a**) IPA canonical pathway *Inhibition of Angiogenesis by TSP1* was predicted to be activated. (**b**) IPA canonical pathway *EIF2 Signalling* was predicted to be inhibited. (*) Yellow arrows indicate findings inconsistent with the state of downstream molecule. (**c**) Significantly altered IPA canonical pathways without predicted activation state. The pathways in **(a)** and **(b)** were generated through the use of IPA (QIAGEN Inc., https://www.qiagenbioinformatics.com/products/ingenuity-pathway-analysis) [1].

Supplementary Figure S2. Wnt/β-catenin–signalling. (**a**) IPA canonical pathway *Wnt/β-catenin–signalling* was predicted to be activated. (*) Yellow arrows indicate findings inconsistent with the state of downstream molecule. (**b**) Heatmap of log_2-transformed normalized read counts of DEGs in the IPA canonical pathway *Wnt/β-catenin–signalling*. The pathway in **(a)** was generated through the use of IPA (QIAGEN Inc., https://www.qiagenbioinformatics.com/products/ingenuity-pathway-analysis) [1].

Supplementary Figure S3. Overrepresented functions related to lymphatic development. (**a**) Network of DEGs based on which IPA function *Migration of lymphatic system cells* was predicted to be activated. (**b**) Network of DEGs based on which IPA function *Proliferation of lymphatic endothelial cells* was predicted to be activated. (*) Yellow arrows indicate findings inconsistent with the state of downstream molecule. The networks in **(a)** and **(b)** were generated through the use of IPA (QIAGEN Inc., https://www.qiagenbioinformatics.com/products/ingenuity-pathway-analysis) [1].

Supplementary Figure S4. Wounding and inflammatory response –related GO-term and IPA function overrepresentation. (**a**) Heatmap of log_2-transformed normalized read counts of DEGs in the GO-term *Response to wounding*. (**b**) Heatmap

of log_2-transformed normalized read counts of DEGs in the GO-term *Inflammatory response*. (**c-e**) Networks of DEGs based on which functions *Leukocyte migration* (c), *Binding of leukocytes* (d) and *Cell movement of mononuclear leukocytes* (e) were significantly increased in IPA analysis. (*) Yellow arrows indicate findings inconsistent with the state of downstream molecule. The networks in **(c)**, **(d)** and **(e)** were generated through the use of IPA (QIAGEN Inc., https://www.qiagenbioinformatics.com/products/ingenuity-pathway-analysis) [1].

Supplementary Figure S5. RPE cell signature genes upregulated in RRD-PVR. (**a**) Bar chart showing top ten GO biological processes, cellular components and molecular functions as well as a KEGG pathway enriched based on DEGs downregulated in PDR, i.e. upregulated in RRD-PVR. (**b**) Heatmap of log_2-transformed normalized read counts of DEGs among the RPE cell signature genes [2].

Supplementary Figure S6. Epithelial to mesenchymal transition involved in PDR and RRD-PVR. (**a**) A portion of the IPA canonical pathway *Regulation of the EMT.* The activation of the function *EMT* was predicted to occur through TGF-β signaling and SMAD2, -3 and -4 activation. (**b**) Network of DEGs based on which IPA function *EMT* was predicted to be activated. (**c**) IPA canonical pathway *TGF-β signaling* was predicted to be activated. (**d**) Network of DEGs based on which function of TGF-β1 as an upstream regulator was predicted to be activated in IPA upstream regulator analysis. (*) Yellow arrows indicate findings inconsistent with the state of downstream molecule. The network and pathways in **(a)**, **(b)**, **(c)** and **(d)** were generated through the use of IPA (QIAGEN Inc., https://www.qiagenbioinformatics.com/products/ingenuity-pathway-analysis) [1].

Supplementary Figure S7. LungGENS signature genes. Heatmaps of $\log_2$-transformed normalized read counts of DEGs among the (**a**) “pericyte”, (**b**) “myofibroblast”, (**c**) “matrix fibroblast” and (**d**) “vascular smooth muscle cell” signature genes, retrieved from the Lung Gene Expression iN Single-cell (LungGENS) database [3].

Supplementary Table Legends

Supplementary Table S1. The demographics of PDR and RRD-PVR patients.

PDR, proliferative diabetic retinopathy; RRD, rhegmatogenous retinal detachment; PVR, proliferative vitreoretinopathy; SD, standard deviation; N, total number of patients; BMI, body mass index; ASA classification, American Society of Anesthesiologists Physical Status classification; NA, not available; VEGFA, vascular endothelial growth factor A; OCT, optical coherence tomography. *, Data not available on one or more patients.

Supplementary Table S2. Individual PDR and RRD-PVR patient systemic and ocular characteristics.

ID, identification number; PDR, proliferative diabetic retinopathy; RRD, rhegmatogenous retinal detachment; PVR, proliferative vitreoretinopathy; SD, standard deviation; BMI, body mass index; ASA classification, American Society of Anaesthesiologists Physical Status classification; VEGFA, vascular endothelial growth factor A; OCT, optical coherence tomography. NA, data not available; (*), patient belongs to the previously described 2011-2017 cohort [4]; (†) 0, no; 1, yes; (‡) Ranibizumab, 2 injections, latest 6 months 21 days prior to operation; (§) 0, none; 1, partial panretinal photocoagulation; 2, total panretinal photocoagulation.

Supplementary Table S3. DESeq analysis and normalized read counts. (**A**) Normalized read counts of all genes. (**B**) All differentially expressed genes. (**C**) All differentially expressed genes upregulated in PDR. (**D**) All differentially expressed genes downregulated in PDR.

Supplementary Table S4. Principal component analysis (PCA) results. (**A**) PC1. (**B**) PC2. (**C**) PC3. (**D**) Biological processes enriched based on the top 50 genes in PC1. (**E**) Biological processes enriched based on the top 50 genes in PC2. (**F**) Biological processes enriched based on the top 50 genes in PC3.

Supplementary Table S5. GO-terms and KEGG pathways based on upregulated DEGs. (**A**) Biological processes. (**B**) Cellular components. (**C**) Molecular functions. (**D**) KEGG pathways.

Supplementary Table S6. IPA results. (**A**) Canonical pathways. (**B**) Upstream regulators. (**C**) Regulator effects. (**D**) Functions.

Supplementary Table S7. GO-terms and KEGG pathways based on downregulated DEGs. (**A**) Biological processes. (**B**) Cellular components. (**C**) Molecular functions. (**D**) KEGG pathways.

References

1 Krämer, A., Green, J., Pollard, J., Jr. & Tugendreich, S. Causal analysis approaches in Ingenuity Pathway Analysis. *Bioinformatics (Oxford, England)* **30**, 523-530, doi:10.1093/bioinformatics/btt703 (2014).

2 Strunnikova, N. V. *et al.* Transcriptome analysis and molecular signature of human retinal pigment epithelium. *Human molecular genetics* **19**, 2468-2486, doi:10.1093/hmg/ddq129 (2010).

3 Du, Y., Guo, M., Whitsett, J. A. & Xu, Y. 'LungGENS': a web-based tool for mapping single-cell gene expression in the developing lung. *Thorax* **70**, 1092-1094, doi:10.1136/thoraxjnl-2015-207035 (2015).

4 Gucciardo, E. *et al.* The microenvironment of proliferative diabetic retinopathy supports lymphatic neovascularization. *The Journal of pathology* **245**, 172-185, doi:10.1002/path.5070 (2018).
